# Multiple conformations of trimeric spikes visualized on a non-enveloped virus

**DOI:** 10.1038/s41467-022-28114-0

**Published:** 2022-01-27

**Authors:** Yinong Zhang, Yanxiang Cui, Jingchen Sun, Z. Hong Zhou

**Affiliations:** 1 Subtropical Sericulture and Mulberry Resources Protection and Safety Engineering Research Center, Guangdong Provincial Key Laboratory of Agro-animal Genomics and Molecular Breeding, College of Animal Science, South China Agricultural University, Guangzhou, Guangdong, 510642 China; 2 California Nanosystems Institute, University of California, Los Angeles (UCLA), Los Angeles, CA 90095 USA; 3 Department of Microbiology, Immunology and Molecular Genetics, UCLA, Los Angeles, CA 90095 USA

**Keywords:** Cryoelectron microscopy, Virus structures

## Abstract

Many viruses utilize trimeric spikes to gain entry into host cells. However, without in situ structures of these trimeric spikes, a full understanding of this dynamic and essential process of viral infections is not possible. Here we present four in situ and one isolated cryoEM structures of the trimeric spike of the cytoplasmic polyhedrosis virus, a member of the non-enveloped *Reoviridae* family and a virus historically used as a model in the discoveries of RNA transcription and capping. These structures adopt two drastically different conformations, closed spike and opened spike, which respectively represent the penetration-inactive and penetration-active states. Each spike monomer has four domains: N-terminal, body, claw, and C-terminal. From closed to opened state, the RGD motif-containing C-terminal domain is freed to bind integrins, and the claw domain rotates to expose and project its membrane insertion loops into the cellular membrane. Comparison between turret vertices before and after detachment of the trimeric spike shows that the *trimeric* spike anchors its N-terminal domain in the iris of the *pentameric* RNA-capping turret. Sensing of cytosolic S-adenosylmethionine (SAM) and adenosine triphosphate (ATP) by the turret triggers a cascade of events: opening of the iris, detachment of the spike, and initiation of endogenous transcription.

## Abstract (web summary)

Zhang and Cui et al. present in situ cryoEM structures of the trimeric spike of cytoplasmic polyhedrosis virus in both open and close conformations, and demonstrate that spike detachment from the capsid is triggered by the presence of SAM and ATP.

## Introduction

The devastating COVID-19 pandemic has brought public awareness to the need to fully understand how viral spikes hide and engage their receptor-binding motifs to enable viral spread. Many viruses, whether enveloped or non-enveloped, use stealthy trimeric spikes with multiple conformations to facilitate cell entry^1^. Similar to many enveloped viruses—notably the influenza^2^, human immunodeficiency^3^ and SARS-CoV-2 viruses^4–6^—some non-enveloped viruses in the *Reoviridae* use a trimeric surface spike protein complex for cell entry^7–11^. For some enveloped^2,3,12–18^ and non-enveloped viruses^9,10,19,20^, structures of spike trimers in isolation have been determined, including several for both pre- and post-fusion conformations^12–19^. Recently, structures of the spike trimers on the native SARS-CoV-2 virion^4–6^ and on the recoated rotavirus triple-layered particles^11^ have been resolved, revealing their drastically different cell entry mechanisms. However, high-resolution in situ structures in multiple states have remained unavailable for non-enveloped viral spike trimers, preventing comparison of these states and consequently a complete understanding of the extremely dynamic process of cell entry.

As a single-shelled member of the *Reoviridae*, the cytoplasmic polyhedrosis virus (CPV) has historically been used as a model in the discovery of RNA transcription and capping^21–26^. CPV has two functionally essential components located along the icosahedral 5-fold axis: the RNA-dependent RNA polymerase (RdRp) inside^23,26^ and the RNA capping turret outside;^25,27^ a trimeric spike is attached to the latter^28^. The external location of this spike implies that it is the receptor-binding and membrane penetration protein^29^. However, previous structures of CPV did not even reveal the oligomeric (trimeric or otherwise) nature^27,30^, let alone conformational states, of the receptor-binding and membrane-penetration spike, due to symmetry mismatch and possible conformational heterogeneity of the spike complex with the underlying pentameric turret. Thus, even though several potential receptors, including sialic acid^31^, glycosylphosphatidylinositol-anchored alkaline phosphatase^29^, and integrin^32^, have been shown to participate in the CPV cell-entry process through the clathrin-mediated endocytosis^33^, how CPV utilizes its structural proteins to facilitate the virion’s entry into the host cell remained unknown.

## Results

To bridge that knowledge gap, we imaged CPV particles prepared in the absence of S-adenosylmethionine (SAM) and adenosine triphosphate (ATP) (quiescent CPV, or q-CPV), or presence of both SAM and ATP (SA-CPV) and employed a sub-particle reconstruction workflow^26^ to obtain five near-atomic resolution structures: three different conformations of the trimeric spike, either on (captured from the q-CPV sample) or detached from the virus (captured from the SA-CPV sample); and two structures of the turret vertices, before (captured from the q-CPV sample) and after the trimeric spike detaches (captured from the SA-CPV sample). These structures reveal two distinct conformations of the trimeric spike, which were captured from the same CPV sample (q-CPV): closed conformation and opened conformation, representing the putative penetration-inactive and penetration-active states. These two conformations explain how CPV attaches to the host cell surface and penetrates the endosomal membrane by sequentially exposing the functional components we discovered on the trimeric spike. In addition, the structures of the turret vertices before and after trimeric spike detachment illustrate how the virus senses cytosolic SAM and ATP and detaches the trimeric spikes, allowing the release of newly synthesized mRNA.

### In situ structures of the trimeric spike in closed and opened conformations

**Fig. 1:**
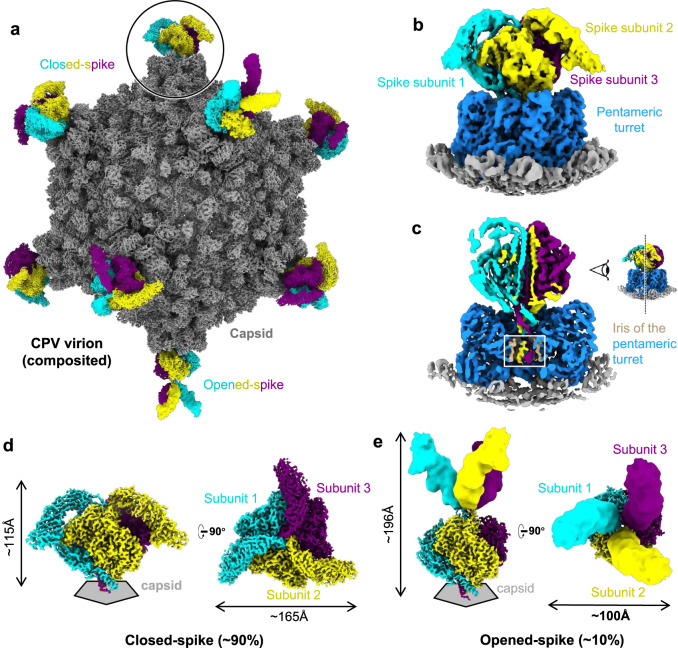
Overall structures of the closed and opened conformations of the CPV trimeric spike. **a** Composite density maps of the capsid (gray) and the trimeric spikes (color) of CPV. Trimeric spikes are colored based on different subunits. **b**, **c** Sub-particle reconstruction (C1) of the trimeric spike interacting with the pentameric turret (blue). Half of the structure is clipped in (**c**) to show interactions. **d**, **e** Side and top views of the sub-particle reconstruction (C3) of the trimeric spikes in closed conformation (**d**) and opened conformation (**e**). The poor resolution parts in (**e**) are filtered for clarity.

To overcome the symmetry mismatch and possible conformational heterogeneity of the trimeric spikes on the CPV capsid, we employed a step-wise symmetry relaxation and sub-particle reconstruction workflow^26^. After obtaining a near-atomic resolution icosahedral reconstruction, we extracted 12 sub-particles from each CPV particle image, i.e., one from every vertex based on the icosahedral orientation of the virus particle. 3D classification of the extracted sub-particles yielded two distinct conformations of the spike structure: closed and opened (Fig. 1 and Supplementary Movie 1).

**Fig. 2:**
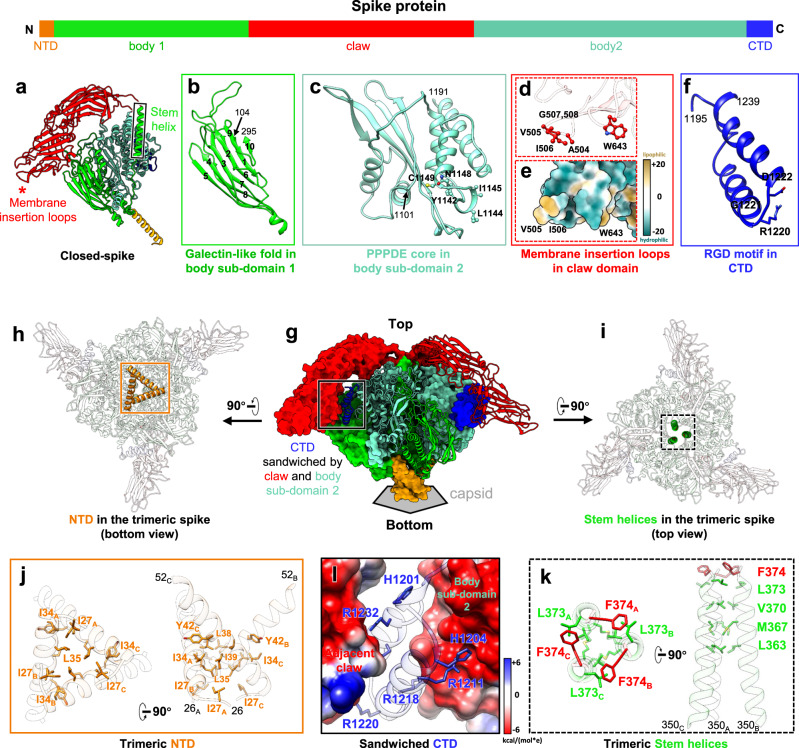
Atomic model and inter-subunit interactions of trimeric spike in closed conformation. **a** Ribbon model of the closed spike monomer, with different domains in different colors. **b–f** Magnified views of the different functional regions of the spike protein, showing the galectin-like fold in the body sub-domain 1 (**b**), the PPPDE core in the body sub-domain 2 (**c**), the hydrophobic membrane insertion loops in the claw domain (**d**, **e**), and the RGD motif in the C-terminal domain (CTD) (**f**). **g**–**i** Ribbon models show the overall structures of the CPV trimeric spike in different views. Two subunits of the trimeric spike in **g** are shown in surface representation. **j**–**l** Magnified views of the boxes in (**g**–**i**). Electrostatic surface renderings in **l** show the negatively charged surface of the claw and body domains, which sandwich the positively charged CTD.

**Fig. 3:**
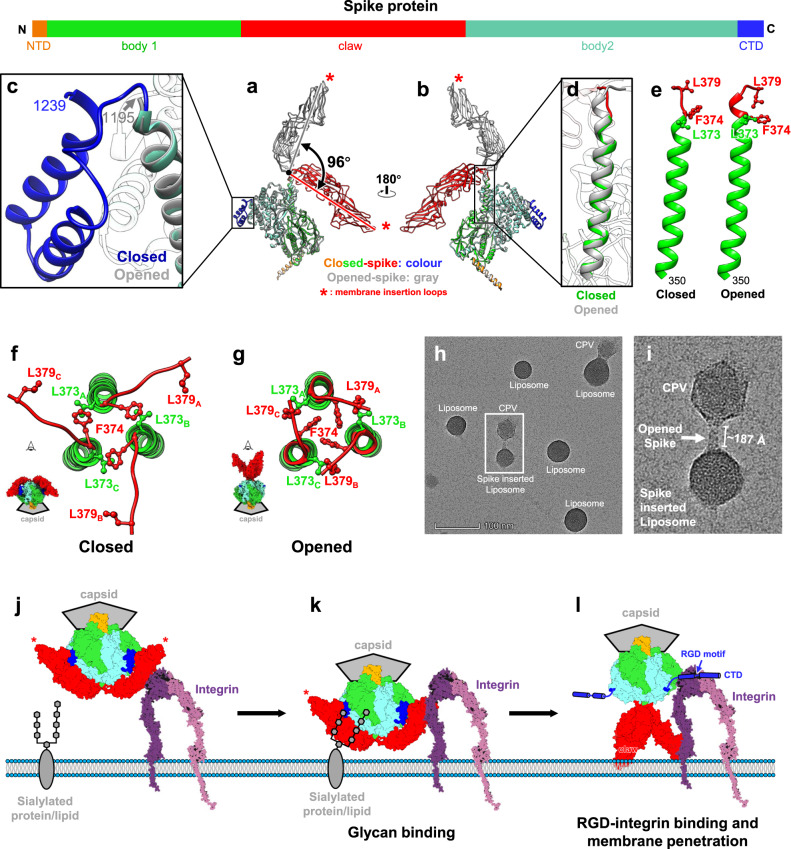
Comparison of the closed and opened spikes. **a**, **b** Superposition of the spike monomer in the closed conformation (color) and opened conformation (gray) in two different views. **c** Magnified view of the boxed region in (**a**). C-terminal domain (CTD), visible (blue) in the closed spike, becomes invisible in the opened spike. **d**, **e** Magnified views of the boxed region in (**b**), showing the conformational changes of the stem-loop in the closed and opened spikes. **f**, **g** Top view of the stem-loops in the trimeric closed (**f**) and opened spikes (**g**). **h**, **i** Representative Negative-stained EM image from two independent experiments shows CPV virions binding to the liposome vesicles. **j**–**l** Schematic diagram illustrating how the trimeric spike interacts with the cell membrane. Before attachment, the trimeric spike of CPV remains in the closed conformation (**j**). In the first step, the sialylated protein/lipid binds to the galectin-like region of the body subdomain 1 (**k**). In the second step, the trimeric spike transforms into opened conformation, the newly revealed RGD motif containing CTD interacts with the integrin, and the exposed membrane insertion loops on the tip of the claw domain insert into the cell membrane (**l**).

From these density maps, we built de novo atomic models for both conformations (Figs. 2 and 3). Protein sequences indicate the spike protein is encoded by the segment 3 gene of *Bombyx mori* CPV-1 (Supplementary Fig. 1). Our atomic models show that the CPV spike complex, like the membrane penetration proteins or fusion proteins of other viruses such as rotavirus^11^ and human immunodeficiency virus^3^, is a homo-trimer (Fig. 1d, e). One trimeric spike is located at an icosahedral 5-fold vertex, interacting with an underlying 5-fold symmetric turret, the RNA-capping enzyme complex (Fig. 1a–c, Supplementary Fig. 2, and Supplementary Movie 1). The root of the trimeric spike plugs into the iris of the pentameric turret like a bottle cork (Fig. 1c). 3D classification of C1 vertex sub-particle reconstructions shows that the trimeric spike and the pentameric turret cannot be resolved in high resolution simultaneously (Fig. 1b, c). This observation indicates that the anchoring orientation of the spike within the turret is non-specific, though the 3-fold axis of the trimeric spike overlaps with the 5-fold axis of the pentameric turret.

### Functional domains and their associated active sites in the trimeric spike of CPV

The atomic model of the spike protein monomer consists of four domains: N-terminal (NTD, aa. 1–52), body (aa. 53–373 and aa. 745–1194), claw (aa. 374–744), and C-terminal (CTD, aa. 1195–1239) (Fig. 2a, Supplementary Figs. 1 and 3).

In our CPV spike structure of both closed and opened conformations, we were unable to model the first 25 residues of the NTD due to poor density, since the N-terminal end, which forms the root of the trimeric spike, is not 3-fold symmetric (Fig. 1c). In the modeled part of NTD, aa. 29–52 form an α-helix (Fig. 2a and Supplementary Fig. 3e), three of which come together from the trimeric spike complex to form a coiled-coil bundle of helices through hydrophobic sidechain interactions (Fig. 2g, h, j).

Above the NTD, the body domain of the trimeric spike is supported by the methylase domains of the turret proteins through electrostatic interactions (Supplementary Fig. 2). The body domain of the monomer contains three β sheets and 23 α helices, including the stem helix (Fig. 2a, Supplementary Fig. 1 and Supplementary Fig. 3f, g). It can be divided into sub-domain 1 (aa. 53–373) and sub-domain 2 (aa. 745–1194), whose amino-acid sequences are separated by that of the claw domain (Supplementary Fig. 1). The two β sheets (aa 104–295) of body sub-domain 1 form a galectin-like fold (Fig. 2b). Body sub-domain 2 contains mainly α-helices (Supplementary Fig. 3g). Aa. 1101–1191 share the general architecture and key residues of the “after permuted papain fold peptidases of dsRNA viruses and eukaryotes” (PPPDE) superfamily (Fig. 2c), as previously predicted^34^.

In the trimer, the stem helix of the body sub-domain 1 of one subunit interacts with the stem helices of the other two subunits. At the interface region of these three stem helices are predominantly hydrophobic residues, including two leucine residues, typical of coiled-coil helix bundles (Fig. 2i, k). These three stem helices of the trimer are surrounded by six α helices of the three body sub-domains 2 in a helix bundle architecture.

At the top of the body domain, the β-strand-rich claw domain connects with the stem helix bundle of the body domain through two loops, one of which interacts with the body sub-domain 1 (aa. 374–379) and the other of which interacts with the body sub-domain 2 (aa. 740–744) (Fig. 2a, g and Supplementary Fig. 3h). The claw domain of the CPV spike and the globulin domain of rotavirus VP5*^19^ both contain the globulin-like fold as well as the putative membrane insertion loops (aa. 503–510 and 640–645) (Fig. 2d, e and Supplementary Fig. 4). However, the CPV claw domain has an additional tip, making the CPV claw domain (102 Å) much longer than that of the rotavirus (75 Å) (Supplementary Fig. 4).

The final part of the monomer, the CTD, is a helix-turn-helix (Fig. 2f and Supplementary Fig. 3i). Within the trimeric spike of CPV, it is sandwiched by the body sub-domain 2 of the same subunit and the claw domain of the adjacent subunit through complementary surface charge (Fig. 2g, l).

### Conformational changes between closed and opened spike

In both the closed and opened conformations of the trimeric spike, the NTD and body domain remain the same (Fig. 3a, b). Additionally, most protein–protein interactions among the three subunits occur on the NTD and the body domain (Fig. 2g–l), indicating that these two domains maintain the conformational integrity of the trimeric spike. By contrast, the conformation of the other two domains, claw and CTD, changes between closed and opened spike (Fig. 3a–c). In the closed spike, the tip of the claw domain that contains the membrane fusion loops points toward the capsid direction, with the RGD-containing CTD visible, though covered (Fig. 2g, l, Supplementary Fig. 3a and Supplementary Movie 2); in the opened spike, the claw domain rotates ~96° and points away from the capsid, while the CTD becomes likely flexible, as it becomes invisible in our structure (Fig. 3a–c and Supplementary Movie 2). Alongside this dramatic rotation of the entire rigid body claw domain, the loop between aa. 374–376 at the beginning of the claw domain refolds into a helix turn, slightly lengthening the helix (Fig. 3d, e). In the three elongated stem helices, the hydrophobic residues L373, F374, and L379 create a hydrophobic core above the body domain: the F374 residues twist towards the 3-fold axis of the trimeric spike and interact with each other through T-shaped π-π stacking interactions; the L379 residue replaces the F374 residue in interacting with the L373 residue of the adjacent subunit (Fig. 3f, g). These conformational changes reduce the length of the stem helix-connecting loop of the claw domain, change the direction of the claw domain, and stabilize the opened spike, thus exposing the membrane insertion loops, which face away from the capsid (Fig. 3a, b and Supplementary Movie 2).

To explore whether the opened spike interacts with the membrane through the exposed claw domain, we carried out a virion-liposome binding assay. Negative stained EM images show that CPV attaches to the liposomal vesicle through a column-like component. The lengths of these components (151–187 Å) are clearly longer than the length of the closed spike (~115 Å) and closer to the length of the opened spike (~196 Å) (Fig. 3h, i and Supplementary Fig. 5), suggesting that these column-like components might be the opened spikes and can interact with liposome vesicles through the exposed membrane insertion loops. We thus speculate that the closed spike represents the putative penetration-inactive state, which, through its galectin-like region, might interact with the sialic acid moiety on the cell membrane^31^ (Fig. 3j, k and Supplementary Fig. 6), while the opened spike represents the putative penetration-active state, which might bind integrin through its RGD motif and penetrate the cell membrane through its membrane insertion loops (Fig. 3l).

Though we observed the two different conformations of the trimeric spike, a question remains: how do these drastically conformational changes occur? To explore whether pH has a role, we incubated CPV under low and high pH conditions (see “Methods”), and reconstructed the in situ structures of the trimeric spikes with our sub-particle reconstruction workflow. We found that the ratio between the open and closed conformations of the spike did not change under either condition, suggesting that the pH change is not the trigger of conformational changes of the spike. This observation also implies that the different conformations of the membrane fusion/penetration proteins always exist on the same virus particle in nature, like in rotavirus^11^ and SARS-Cov-2^4,6^.

### Presence of SAM and ATP triggers detachment of trimeric spike from pentameric turret

**Fig. 4:**
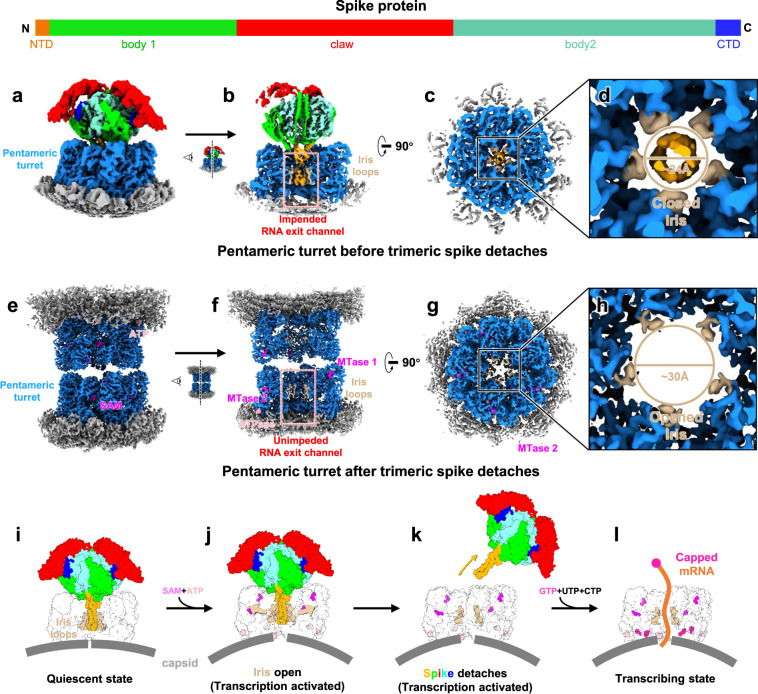
Comparison of the pentameric turrets before and after the trimeric spike detaches. **a**, **b** Sub-particle reconstruction (C1) of the pentameric turret before the trimeric spike detaches. Half of the structure is clipped in (**b**) to show spike-turret interactions and the impeded RNA exit path. **c** Clipped top view shows the “cork” formed by the N-terminal domains (NTDs) of the trimeric spike plugging into the closed iris of the pentameric turret. **d** Magnified view of the boxed region in (**c**). **e**, **f** Sub-particle reconstruction (C5) of the two spike-free pentameric turrets interacting with each other. Half of the structure is clipped in (**f**) to show the unimpeded RNA exit path. **g** Clipped top view shows the opened iris of the pentameric turret. **h** Magnified view of the boxed region in (**g**). **i**–**l** Schematic diagram illustrates how CPV regulates the attachment/detachment of the trimeric spike. In the quiescent state, the pentameric turret’s iris loops hold the trimeric spike tightly, and the turret’s RNA exit path is impeded by the trimeric spike (**i**). When the turret proteins sense SAM and ATP, the iris opens as the turret proteins change conformations (**j**); the trimeric spike thus easily detaches from the virion (**k**), allowing the mRNA to be capped and released through the turret’s RNA exit path (**l**).

In the turreted subfamily of the *Reoviridae*, represented by CPV, the turret protein is a multi-functional protein: it serves as the capping enzyme, the signal sensor, and the mRNA releasing channel on the five-fold axis of the capsid^25–27^. In our structure of the pentameric turret prior to detachment of the trimeric spike, the NTDs of the trimeric spike “cork” the turret iris, which is formed by loops (aa. 505–518), and impedes the mRNA releasing channel (Fig. 1c and Fig. 4a–d). To determine how CPV releases new transcripts during transcription, we prepared a CPV sample treated with SAM and ATP-containing buffer (SA-CPV), which has been shown to activate the RdRP for mRNA synthesis^26^. Serendipitously, turrets of some adjacent viral particles “kiss” one another in our structure, connecting their open ends and forming a dimer (Fig. 4e and Supplementary Fig. 7a, b). In this dimer, there is no room for the trimeric spike to attach inside the turret; therefore, we concluded that in these structures, the trimeric spike has thoroughly detached (Fig. 4e–h and Supplementary Fig. 7a, b). Additionally, we extracted free spikes already detached from the virions, visible in the background of the SA-CPV micrographs. The reconstruction result shows that the majority of these particles are in closed spike conformation (Supplementary Fig. 7c), indicating that the conformational change of the trimeric spike was not caused by spike detachment from the virion.

Comparison of sub-particle reconstructions of turrets before and after spike detachment shows that the binding of SAM and ATP to the turret protein causes global conformational changes (Supplementary Fig. 7d–i). With these conformational changes, the diameter of the iris increases from 24 Å to 30 Å (Fig. 4d, h). The opening of the iris might weaken the interactions between the iris loops and the “cork” of the spike proteins, thus allowing the trimeric spike lodged in the middle of the iris to detach. The detachment of the spike clears the mRNA releasing channel and enables nascent mRNA to reach the methyltransferase (MTase) active sites and finish mRNA capping (Fig. 4b, c, f, g), allowing the mature mRNA to be released outside the capsid (i.e. into the cytosol) (Fig. 4i–l). Therefore, upon sensing cytosolic SAM and ATP, CPV orchestrates the sequential functions of the turret protein through conformational changes, allowing the virus to go through the various steps of viral infection, from host cell invasion to transcription^25,26^ (Supplementary Fig. 8).

## Discussion

The five structures of the CPV trimeric spike presented here, when combined with previous biochemical studies, support a cell-entry process for a non-enveloped virus that is conserved among many enveloped viruses (Supplementary Fig. 8). During native infection, CPV-embedding polyhedra are swallowed by silkworm *Bombyx mori* and dissolved in the mid-gut. The virions released from polyhedra then utilize their surface trimeric spikes to interact with sialic acid moieties on the cell membrane^31^ (Supplementary Fig. 8a, b), and the virions’ turret protein interacts with the host cell’s glycosylphosphatidylinositol-anchored alkaline phosphatase^29^ (Supplementary Fig. 8c). After the virus is internalized by the host cell through clathrin-mediated endocytosis^33^ (Supplementary Fig. 8d), the trimeric spike changes conformation, freeing its CTD; its RGD motif then binds to integrin on the endosomal membrane (Supplementary Fig. 8e). The exposed membrane insertion loops on the claw domain insert into the endosomal membrane, facilitating endosome disruption for viral release into the cytoplasm (Supplementary Fig. 8e–g). Upon sensing cytosolic SAM and ATP, the turret protein changes its conformation, causing the trimeric spike to detach from the turret (Supplementary Fig. 8g) and allosterically controlling initialization of RNA transcription inside the virus^26^ (Supplementary Fig. 8h). Newly synthesized mRNA is capped and released from the turret into the cytoplasm for protein translation, viral assembly, and replication promoted by CPV-encoded PPPDE, ultimately starting a new cycle of infection.

In our structures, we observed an integrin-binding RGD motif located in the CTD of the trimeric spike. Both enveloped viruses^35^ and non-enveloped viruses^36^ have been found to use an RGD motif to attach to the cell membrane or trigger endocytosis to facilitate viral internalization^37^. However, the RGD motif of the CPV trimeric spike is covered in the putative penetration-inactive state (i.e., closed spike) and exposed in the putative penetration-active state (i.e., opened spike), while the integrin-binding motifs known in other viruses are always exposed (Supplementary Fig. 9). Therefore, we speculate that the RGD motif does not trigger the endocytic pathway by interacting with integrin, but rather interacts with integrin after endocytosis and regulates downstream signaling for the virus’ preferred endocytic sorting pathway^38^ to ensure that the virus is endocytosed along the “correct route”.

Viruses utilize viral encoded deubiquitinating enzymes (DUBs) to suppress host innate antiviral responses and promote viral replication^39^. DUBs are always found to be part of a larger viral protein, such as non-structural proteins and tegument proteins^39^. PPPDE has been predicted previously in dsRNA viruses, where they are suggested to have DUB-like functions^34^. In our structures of the CPV trimeric spike, we observed the PPPDE core in the body sub-domain 2, though its active site is buried inside the trimeric spike. Thus, the PPPDE core might be exposed in the monomeric or truncated spike protein, either detached from the viral capsid or expressed by mRNA, and execute its DUB functions inside the cytosol to suppress the innate antiviral responses of the host cell and facilitate CPV replication. Our observation of the PPPDE core-containing spike protein showcases how segmented dsRNA viruses express multi-functional proteins with limited genes to facilitate viral proliferation.

Taken together, our study reveals how CPV, a non-enveloped virus, attaches to the cell surface, penetrates the cell membrane, and “clears” the releasing channel for newly synthesized mRNA to begin a new cycle of viral assembly and spread. Complementing previous structures of CPV^21–23,27,30,40^, this result represents the last piece of the CPV structural “puzzle” that completes the CPV virion life cycle, from viral invasion to transcription, and sheds new light on life cycles and spike structures of non-enveloped viruses in general. Our study supports a non-enveloped virus cell entry pathway involving molecular events surprisingly similar to those occurring during enveloped virus cell entry. The completion of the atomic structure of all components of CPV—a virus already used as a “green” pesticide in agriculture—opens the door for rational engineering efforts for broader human applications, from vaccine development to drug delivery^41,42^.

## Methods

### CPV sample purification

CPV particles were purified as described previously^26,30^. Briefly, BmCPV (*Bombyx mori* CPV-1, isolated from South China Agricultural University in Guangzhou, China) containing polyhedra were treated with 0.2 M Na_2_CO_3_–NaHCO_3_ buffer (pH 10.8) for 60 min, then centrifuged at 10,000 × *g* for 40 min to remove large substances. The supernatant was centrifuged at 80,000 × *g* for 60 min to pellet the CPV virions. The resulting pellets were resuspended in quiescent-buffer (q-CPV sample), SA-buffer (SAM + ATP, SA-CPV sample), low pH buffer (pH 3.4), or high pH buffers (pH 10.8) as previously described^26^. Samples were incubated at 31 °C for 15 min and placed on wet ice at 0 °C prior to cryoEM sample preparation.

### CryoEM sample preparation, image acquisition, and pre-processing

An aliquot of 2.5 μl of each of the above-described samples was applied onto a Quantifoil holey carbon grid (R2/1, 300 mesh), which was glow-discharged for 30 s with a PELCO Easy Glow system. The grid was blotted with filter paper to remove excess sample, then plunge-frozen in a mixture of ethane and propane liquid (mix ratio around 1:2 by volume) with a manual plunger. The frozen grids were stored in liquid nitrogen before use.

CryoEM grids were loaded into an FEI Titan Krios electron microscope equipped with a Gatan imaging filter (GIF) Quantum system and a post-GIF K2 Summit direct electron detector. The microscope was operated at 300 kV, and the GIF slit was set to 20 eV. Movies were recorded as dose-fractionated frames with SerialEM^43^, at ×130,000 nominal magnification in super-resolution mode (corresponding to a calibrated pixel size 0.531 Å on the specimen level), and the defocus was set to −1.0 to −2.3 µm. During data collection, each movie was recorded with an exposure time of 6 s and an accumulated dosage of about 40 electrons/Å^2^, which was fractionated into 30 frames each with 0.2 s exposure time.

Movies were processed with *MotionCor2*^44^ with a sub-frame 5 × 5 and 2× binned (final pixel size 1.062 Å) to generate both dose-weighted (used for final reconstruction) and dose-unweighted (used for manual screening, CTF determination, and particle picking) averaged micrographs. Defocus determination was done with ctffind4.1.10^45^ and particles picking with Ethan^46^. Micrographs with ice contamination or defocus value outside the range −0.8 to −3 µm were discarded. A total of 9422 micrographs of the neutral pH CPV sample and 4521 micrographs of the SA-CPV sample were selected for subsequent in-depth data processing.

### Sub-particle 3D classification and reconstruction of the trimeric spike-turret complex

We followed a sub-particle reconstruction workflow to determine the in situ structures of the CPV trimeric spikes as detailed previously^26^. In brief, for the quiescent CPV particles, we first obtained an icosahedral reconstruction (I2 symmetry) of whole virus particles in Relion 3.0 or Relion 3.1^47^. We took advantage of the I2 reconstructions and extracted 12 vertex sub-particles from all CPV particle images. These sub-particles were used to obtain reconstructions of the spike trimers. 3D classification yielded two conformations (closed and opened) of the trimeric spike. The final maps with C3 symmetry were obtained using Relion 3D refinement with 310,662 sub-particles, resulting in the closed spike map at 2.7 Å resolution, and 35,387 sub-particles for the opened spike map at 3.3 Å resolution; the final map with C1 symmetry was obtained with 69,955 sub-particles for the closed spike interacting with the pentameric turret map at 4.1 Å resolution.

For the SA-CPV particles, we extracted 12 vertex sub-particles from all CPV particle images and the detached spike particles from the background of the EM images. The final maps were obtained using Relion 3D refinement with 3152 sub-particles, resulting in a turret after spike detachment map at 3.7 Å resolution, and with 69,035 particles for the detached spike map at 3.0 Å resolution.

All resolutions reported above are based on Relion’s “gold standard” refinement procedure and the 0.143 Fourier shell correlation criterion. Local resolution was estimated using Resmap^48^.

### Atomic model building, model refinement, and 3D visualization

Protein model building and model refinement were carried out by following a previously detailed procedure^49^. The procedure involves manual modeling with COOT^50^ and real-space refinement with Phenix^51^.

Lacking any previous structures of this spike-trimer, we manually modeled the closed spike trimer de novo with COOT^50^ and with the aid of secondary structure predictions obtained from Phyre2^52^. First, we identified a polypeptide strand with clear densities of amino-acid residues with bulky or long sidechain, such as sidechains of histidine, phenylalanine, tyrosine, and tryptophan, and identified the N- and C-terminals of this strand. This strand was processed with the “map skeleton” utility of COOT to generate a skeleton, then was manually modeled as a Cα trace, one residue at a time, with ‘C-alpha Baton Mode’ based on the skeleton. We then transformed the ‘Baton Atoms’ from the Cα trace into a polyalanine chain with “Cα Zone -> Mainchain”.

For regions formed as secondary structures, we used the “Place Helix Here” and “Place Strand Here” tools to create a poly-alanine α-helix or β-strand in that location. After most of the easily-identified regions were modeled, the rest of the polypeptide strand was manually modeled, starting from the modeled parts with “C-alpha Baton Mode” and transforming them into polyalanine chains with ‘Cα Zone -> Mainchain’, until all the modellable densities were modeled. Next, each of these polyalanine mainchain chains was mutated to the correct sequence with “Simple Mutate” to create a crude atomic model, which was then fit into the cryoEM map with “Real Space Refine Zone” and “Regularize Zone”.

The model of the spike monomer in the closed conformation was used as the template to rebuild the opened conformation of the spike. The models of the NTD and body domain were fitted into the map of the opened spike, and each residue was inspected and modified with “Real Space Refine Zone” and “Regularize Zone” manually. Due to the low resolution of the claw domain, we directly fit the claw domain’s model of the closed spike into the map of the opened spike, and connected loops were modified with “Real Space Refine Zone” and “Regularize Zone” manually. The crude model of the opened spike was then fitted into the cryoEM map with “Real Space Refine Zone” and “Regularize Zone”.

To refine atomic models, we used the “Ramachandran plot” utility in COOT to identify “outliers”; these residues and their surrounding residues were refined with “Regularize Zone” utility in COOT until the Ramachandran outlier value was below 0.1%. These models were further refined in Phenix with “Real space refinement”. After model refinement with Phenix, models were uploaded to the “wwPDB Validation Service” of the wwPDB website, and the atomic models were refined further based on the validation reports produced by the wwPDB. Model statistics are provided in Supplementary Table 1.

Visualization, segmentation of density maps, and generation of Movies were done with UCSF Chimera^53^ and UCSF ChimeraX^54^. Molecular docking prediction was carried out with Autodock Vina^55^.

### Virion-liposome binding assay

Purified BmCPV was suspended in liposome-containing PBS buffer (20% v/v) (pH 8.0) and incubated at 31 °C for 2 h. The reactions were stopped by quenching the samples on wet ice at 0 °C. An aliquot of 5 μl of the resulting sample was applied onto a Quantifoil holey carbon grid (R2/1, 300 mesh), which was glow-discharged for 30 s with a PELCO Easy Glow system. After 1 min incubation to allow sample absorption, the grid was blotted with filter paper to remove excess sample and then stained by 2% uranyl acetate water solution (w/v) for 2 min. The stained grid was then blotted by filter paper and air dried for 2 h. EM images were manually recorded on a Ceta 16 M CMOS camera in an FEI Talos F200S transmission electron microscope operated at 200 kV.

### Reporting summary

Further information on research design is available in the Nature Research Reporting Summary linked to this article.

## Supplementary information

Supplementary Information

Peer Review File

Description of Additional Supplementary Files

Supplementary Movie 1

Supplementary Movie 2

Reporting Summary

## Data Availability

The data that support the findings of this study are available from the corresponding author upon request. Accession codes for deposited maps in the Electron Microscopy Data Bank include those for the sub-particle reconstructed cryoEM density maps: EMD-32504 (in situ structure of the closed spike), EMD- 32505 (in situ structure of the opened spike), EMD-32506 (in situ structure of the trimeric spike interact with pentameric turret), EMD-32507 (in situ structure of the pentameric turret after the trimeric spike detaches), and EMD-32508 (detached spike). Accession codes for atomic models deposited in the Protein Data Bank are 7WHM (closed spike), 7WHN (opened spike), and 7WHP (turret protein after the trimeric spike detaches).
