# Supplementary material for: Multiple conformations of trimeric spikes visualized on a non-enveloped virus
Source: Nat Commun. 2022 Jan 27;13:550. doi: 10.1038/s41467-022-28114-0 (PMC8795420; doi:10.1038/s41467-022-28114-0)
Supplement: Supplementary file 1 — Supplementary Information [file 41467_2022_28114_MOESM1_ESM.pdf]

Supplementary information

Multiple Conformations of Trimeric Spikes Visualized on a Non-Enveloped Virus

Yinong Zhang[1,2,3,4], Yanxiang Cui[2,3], Jingchen Sun[1,4,5,*], Z. Hong Zhou[3,4,*]

[1]Subtropical Sericulture and Mulberry Resources Protection and Safety Engineering Research Center, Guangdong Provincial Key Laboratory of Agro-animal Genomics and Molecular Breeding, College of Animal Science, South China Agricultural University, Guangzhou, Guangdong 510642, China

[2]These authors contributed equally

[3]California Nanosystems Institute, University of California, Los Angeles (UCLA), Los Angeles, CA 90095, USA

[4]Department of Microbiology, Immunology and Molecular Genetics, UCLA, Los Angeles, CA 90095, USA

[5]Lead contact

*Correspondence:

Email: Hong.Zhou@ucla.edu and Cell phone: 1-310-694-7527 (Z.H.Z. for biochemistry, cryoEM and structures) or cyfz@scau.edu.cn (J.S. for CPV virology)

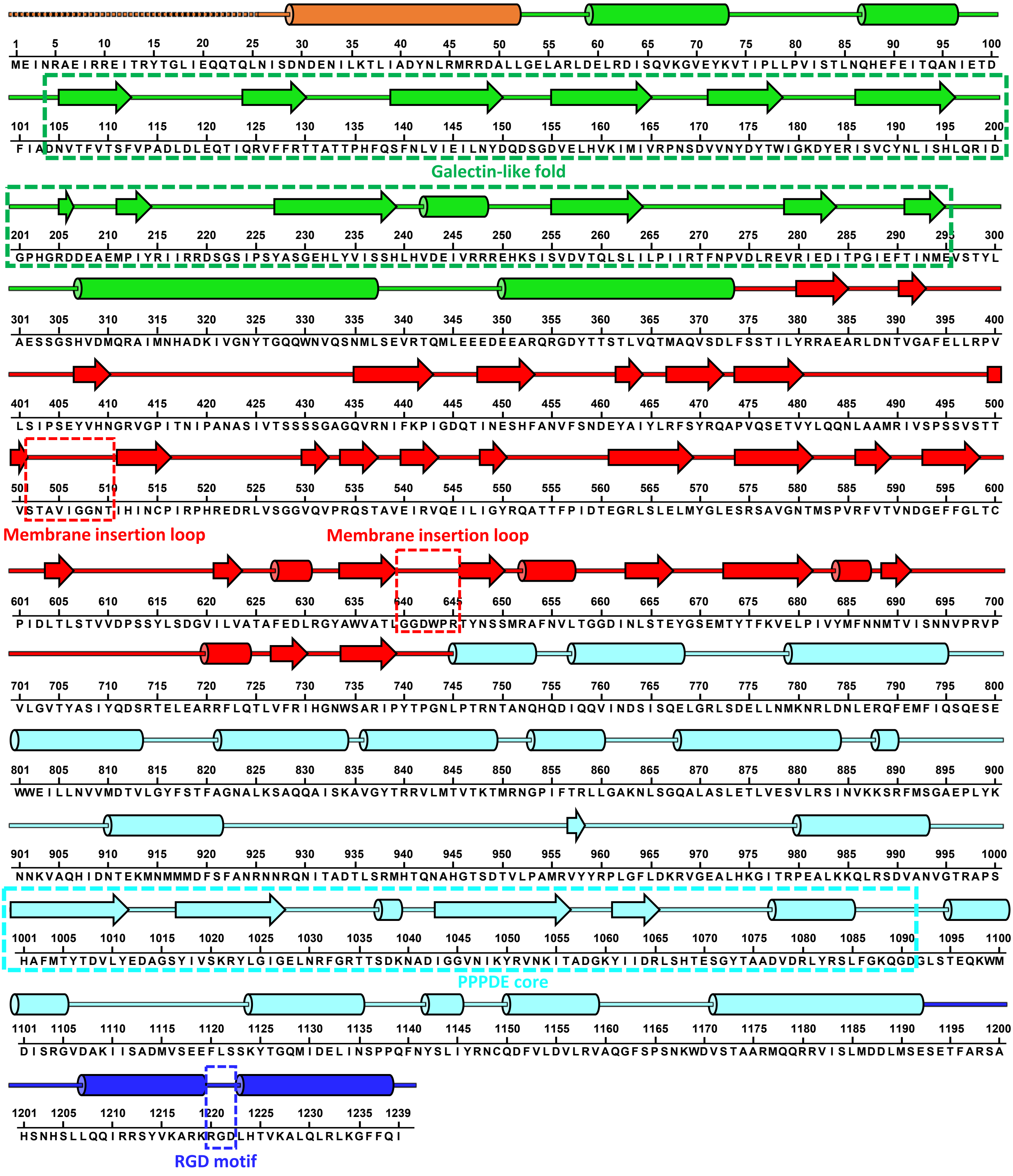

Supplementary Figure 1. Sequence and secondary structure assignment of the spike.

α helices are marked by cylinders, β strands by arrows, loops by thin lines, and the un-modellable part of NTD by dashed lines. Dashed boxes surround sequences of functional regions.

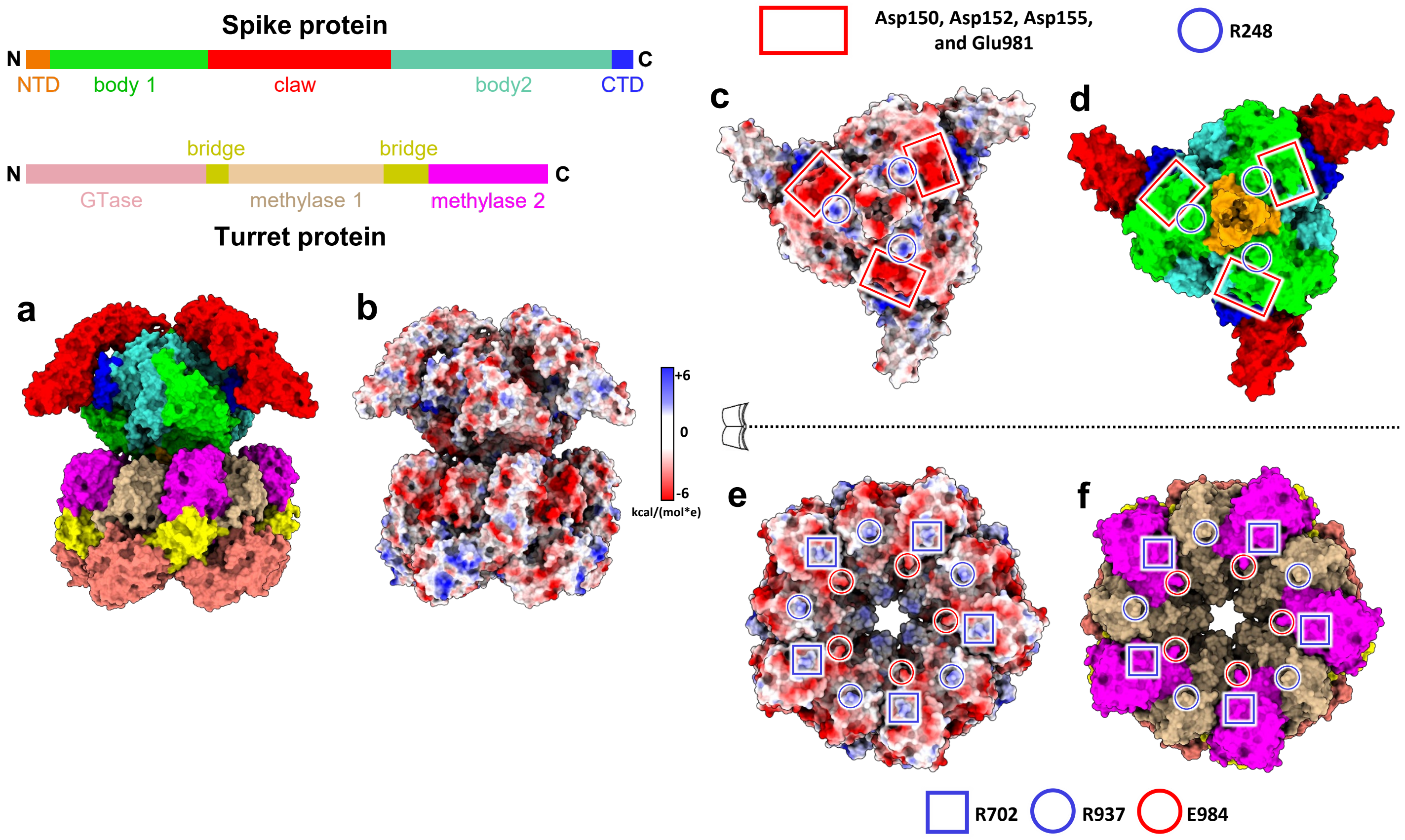

Supplementary Figure 2. Interactions between the trimeric spike and the pentameric turret. **a,b,** Surface views of the atomic structures showing the contact (**a**) and charge (**b**) surfaces between the trimeric spike and the pentameric turret. the colour keys for domains in **a** are shown on top, and those for **b** are next to it. **c-f,** Open book views in **a** and **b** with regions of interest indicated by coloured boxes and circles.

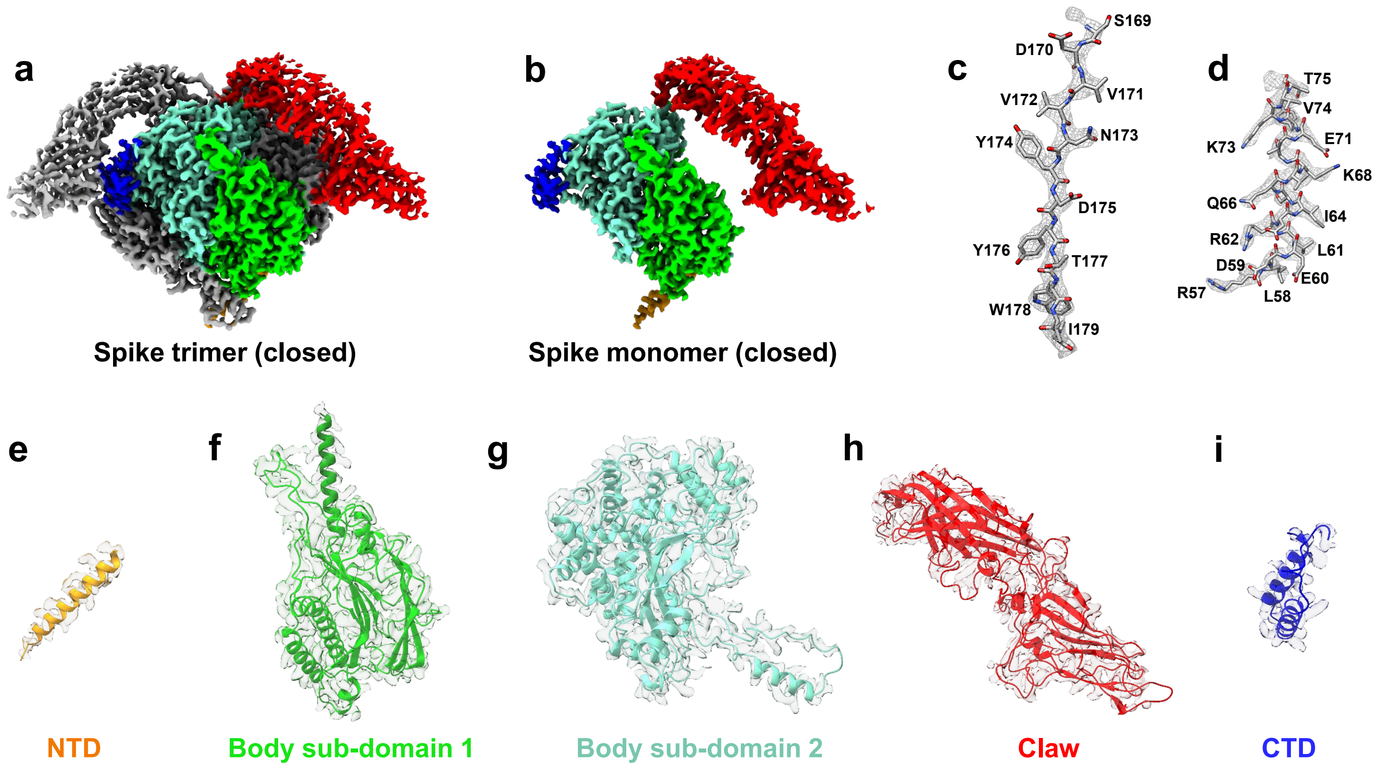

Supplementary Figure 3. Structure of the CPV closed spike.

a, Density map of the closed spike trimer. One subunit of the trimer is coloured based on the subunit's different domains. **b,** Density map of the coloured subunit in **a**. **c,d,** Density maps (mesh) and atomic models (sticks) of a β-sheet (**c**) and an α-helix (**d**) in the closed spike, with residues indicated. **e-i**, Density maps (transparency) and atomic models (sticks) show the different domains of the spike.

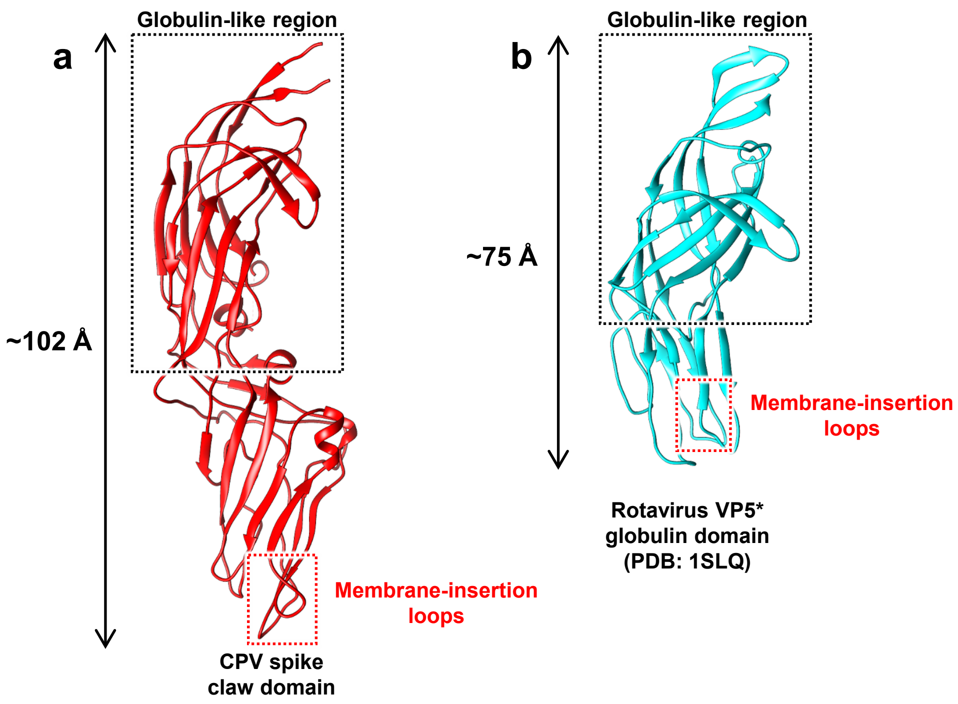

Supplementary Figure 4. Structure comparison of the claw domain in **a**, the CPV spike and **b**, the globulin domain in Rotavirus VP5*.

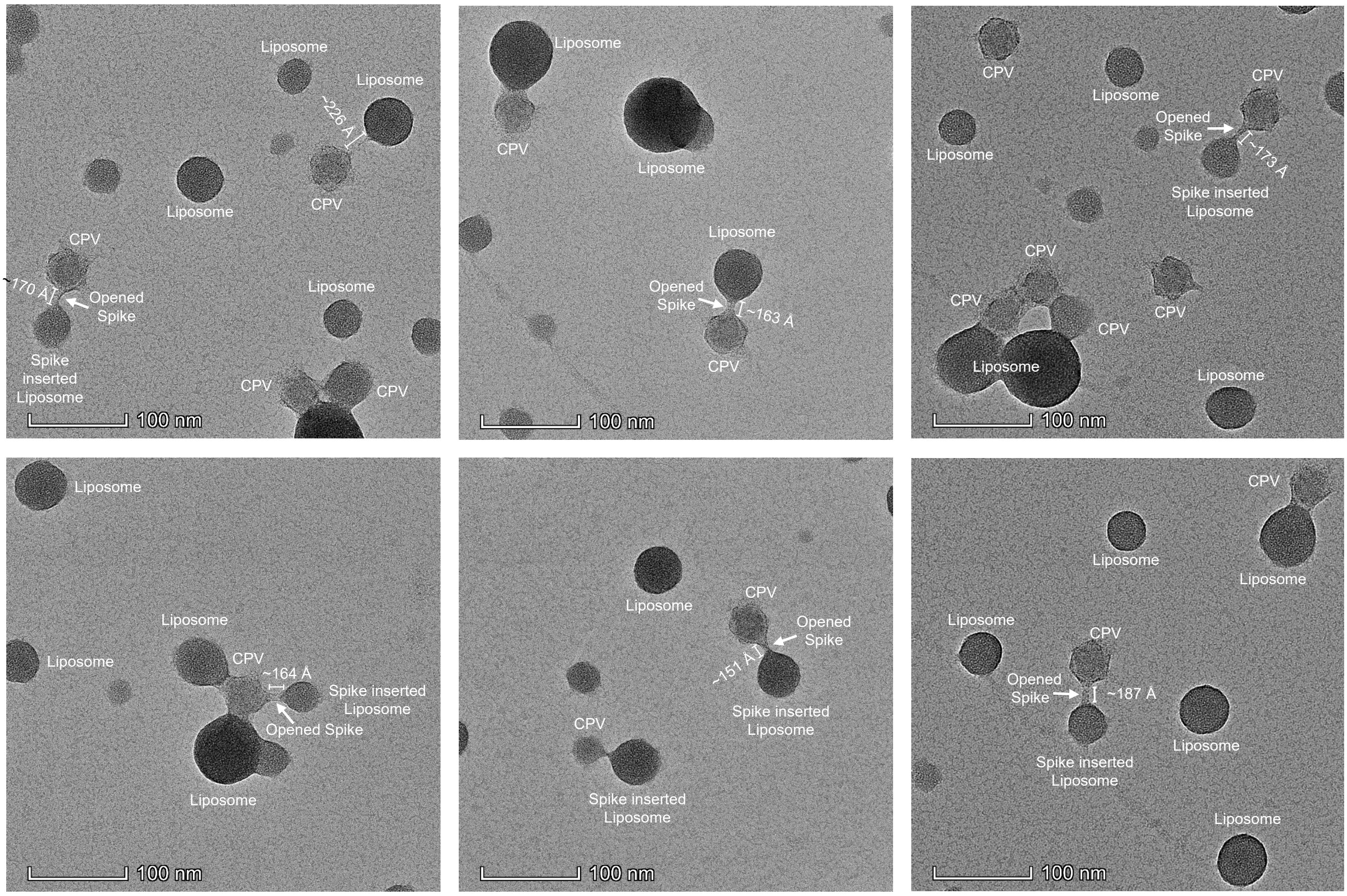

Supplementary Figure 5: Representative Negative-stained EM images from two independent experiments show interactions between the CPV virions and the liposome vesicles.

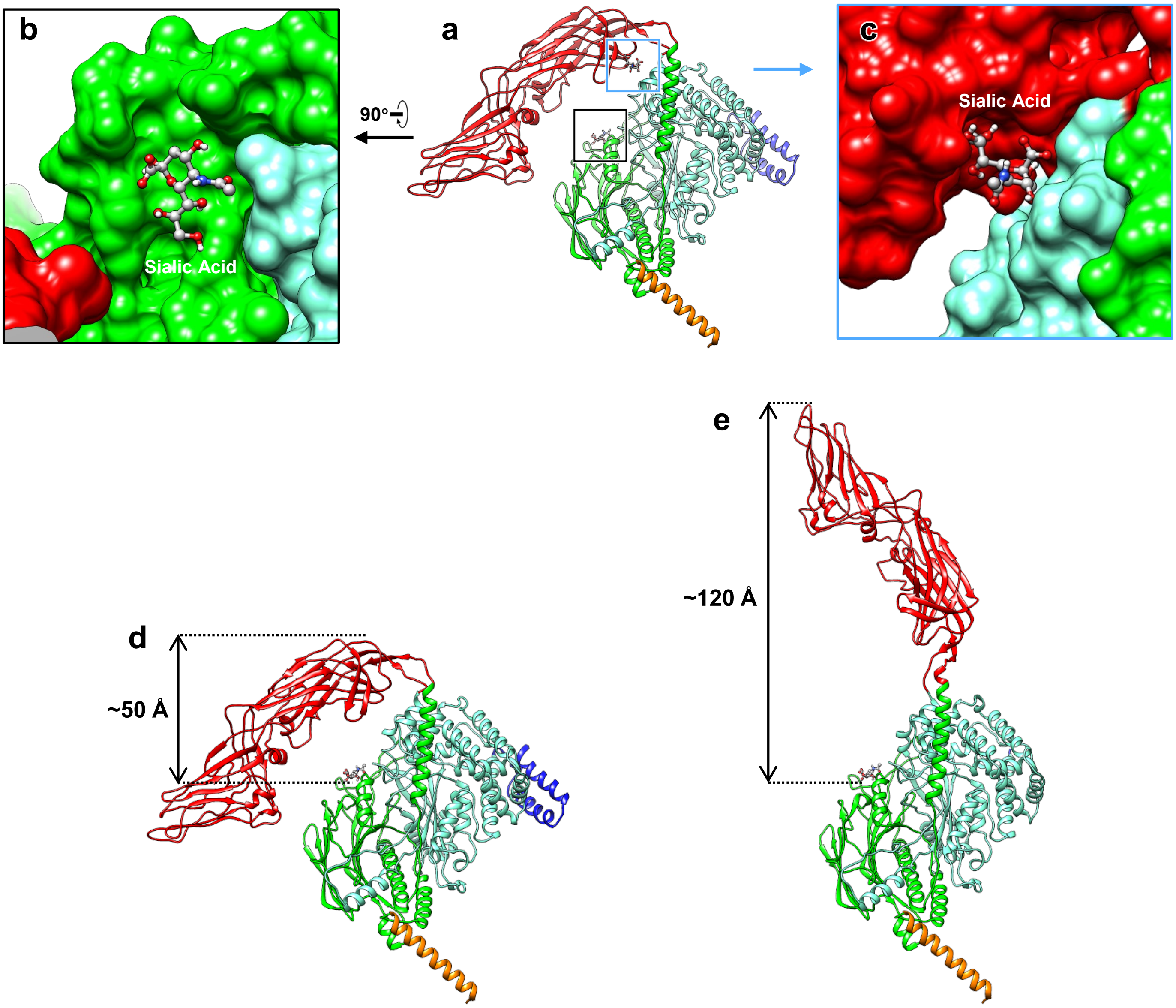

Supplementary Figure 6. Atomic model of sialic acid binding sites of the spike monomer and their respective sialic acid (stick and ball), as predicted by Autodock Vina.

a, Ribbon model of the spike monomer with sialic acids (stick and ball). **b,c**, Magnified views of the two predicted sialic acid binding pockets: one in the body sub-domain 1 (**b**) and the other in the claw domain (**c**). **d,e**, Distances from the predicted sialic acid binding pockets in the body sub-domain 1 to the cell membrane in the spike monomer of CPV in closed (**d**) and opened conformations (**e**).

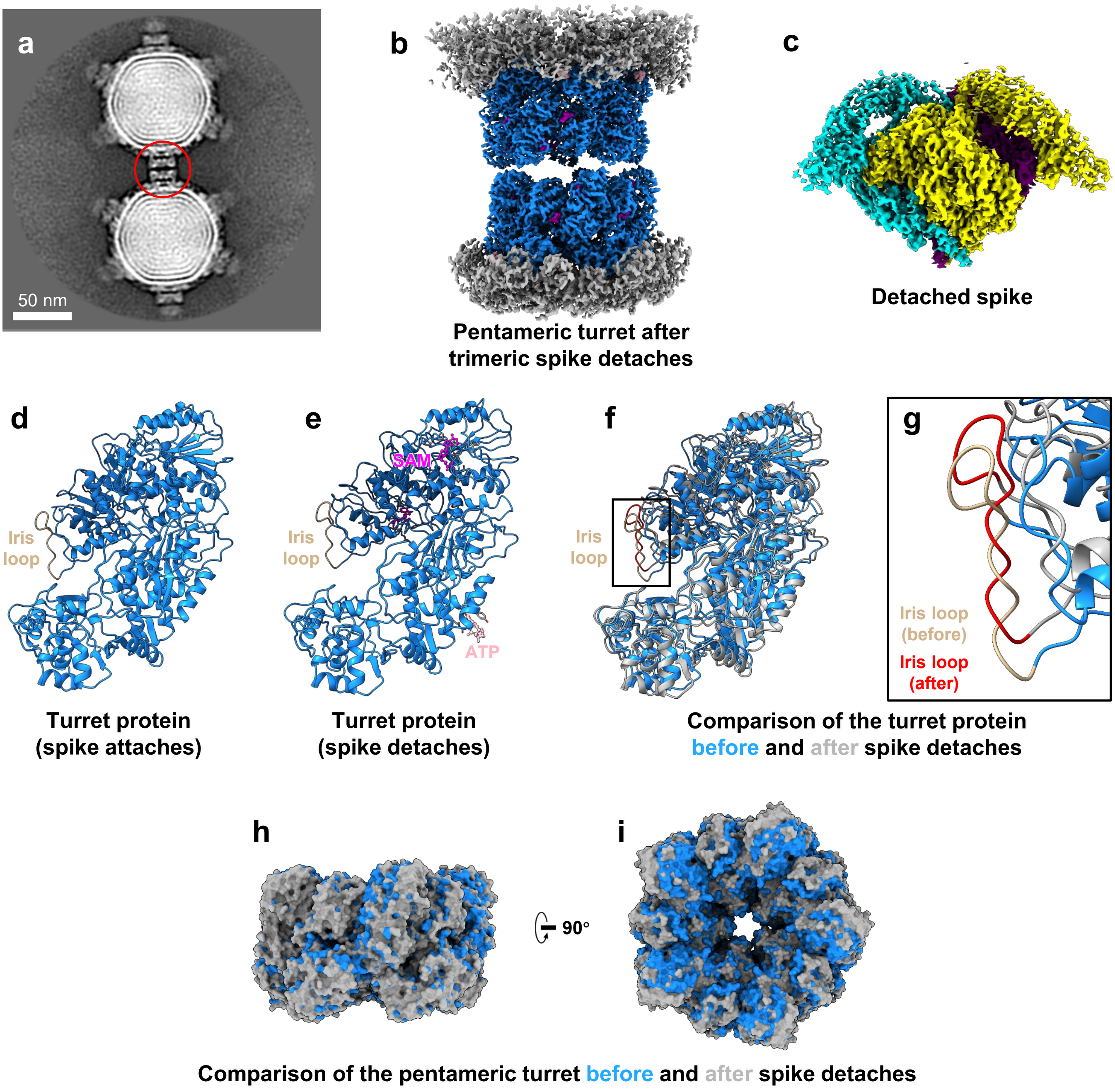

Supplementary Figure 7: 3D reconstructions of the pentameric turret and detached spike in the SA-CPV sample.

a, 2D classification result (3,152 particles in this class) show the two SA-CPV virions "kissing" each other. **b,** Density maps of two "kissing" pentameric turrets, reconstructed from the five-fold vertices of SA-CPV virion. **c**, The detached spike, reconstructed from the background of the cryoEM images of the SA-CPV sample. **d-g,** Ribbon models of the turret protein before (**d**) and after (**e**) detachment of the spike protein, and their comparison (**f,g**). **h,i,** Side (**h**) and top (**i**) views of the comparison between the pentameric turrets (surface representation) before (coloured) and after (gray) detachment of the spike.

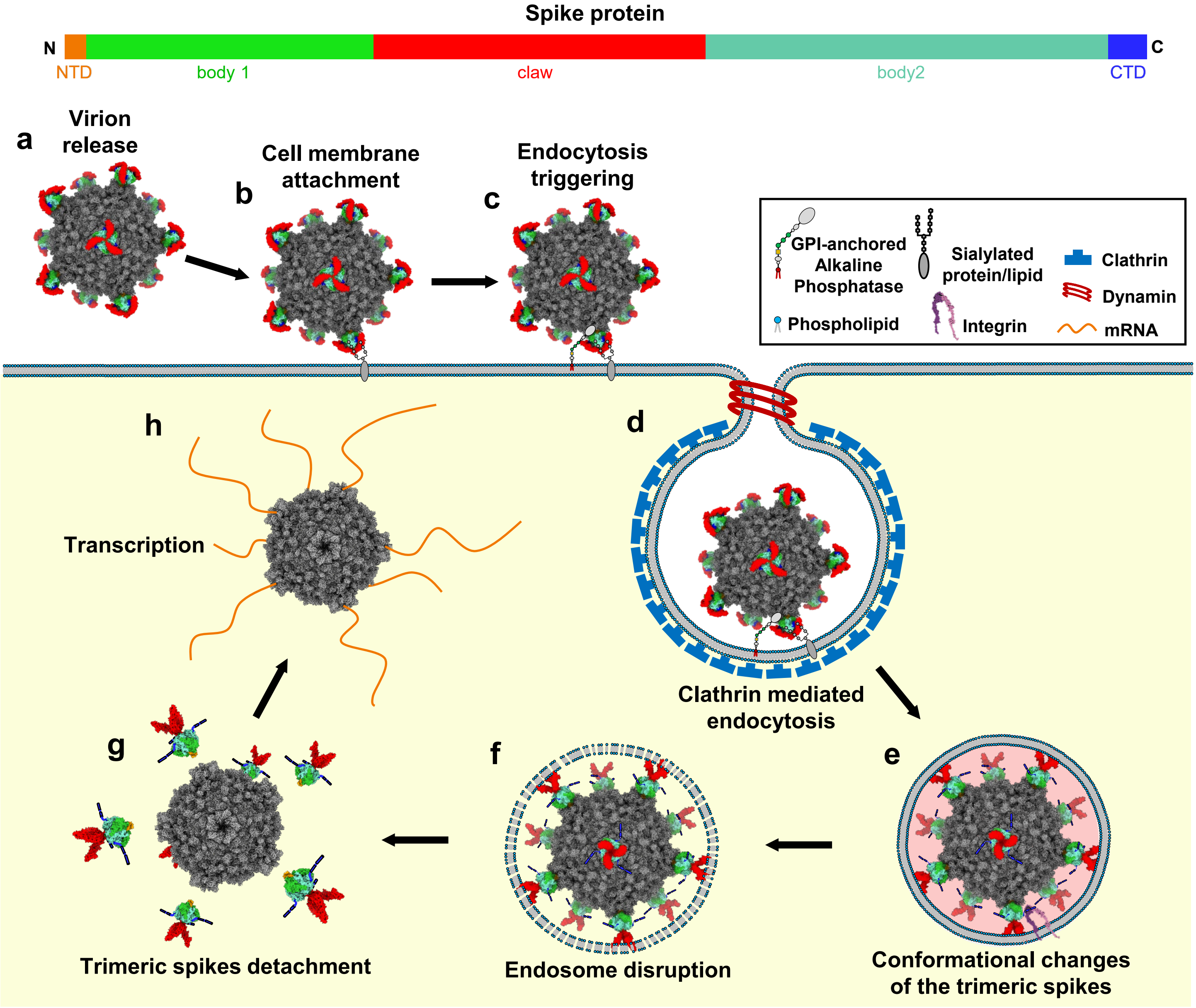

Supplementary Figure 8. Schematic of cell attachment and entry of CPV.
a-h, After the CPV-embedded polyhedral is swallowed by a *Bombyx mori* and dissolved in its mid-gut, the released virions utilize the trimeric spikes on the viral capsid to interact with the sialylated protein/lipid on the host cell membrane (**a**,**b**); the virus' turret proteins then interact with the cell's alkaline phosphatase (**c**). When the CPV is internalized by the host cell through clathrin-mediated endocytosis (**d**), the trimeric spike changes conformations: the RGD motif on the freed CTD becomes able to bind to integrin (**e**), and the newly exposed hydrophobic regions on the claw domain insert into the endosomal membrane, facilitating endosome disruption (**e**,**f**), leading to viral release in the cytoplasm (**g**). Upon sensing cytosolic SAM and ATP, the turret protein changes conformation, causing the trimeric spike to detach from the turret (**g**) and allosterically controlling initialization of RNA transcription inside the virus (**h**).

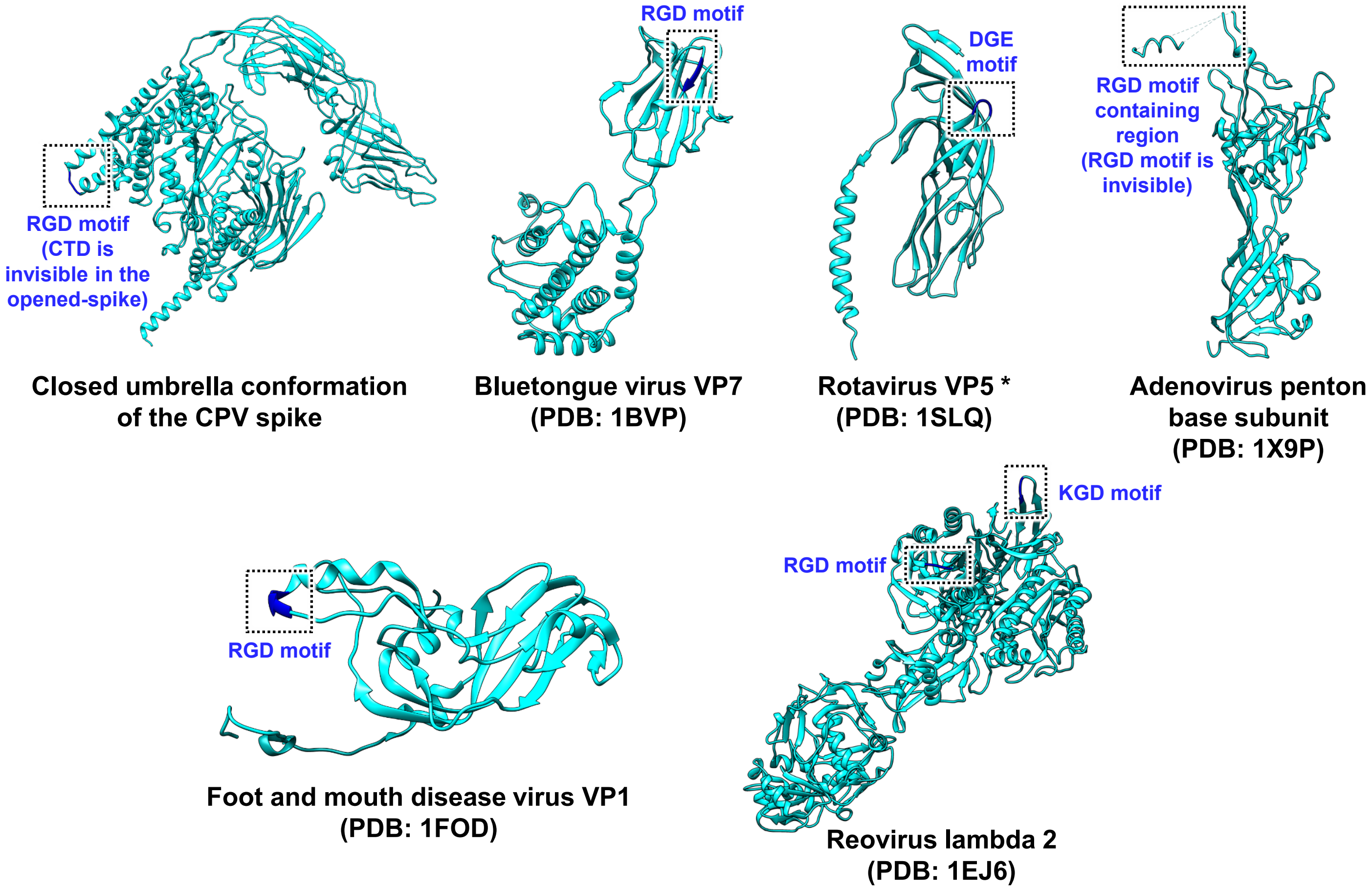

Supplementary Figure 9: Integrin-binding motifs in the structural proteins of non-enveloped viruses.

Supplementary Table 1: Cryo-EM data collection, refinement and validation statistics

Sample	q-CPV			SA-CPV	
Structures	Closed spike (EMD-32504, PDB: 7WHM)	Opened spike (EMD-32505 PDB: 7WHN)	Closed spike interacts with pentameric turret (EMD-32506)	Pentameric turret after spike detaches (EMD-32507 PDB: 7WHP)	Detached closed spike (EMD-32508)
**Data collection**					
Nominal magnification	130,000×				
Microscope	Titan Krios				
Voltage (kV)	300				
Electron exposure (e–/Å^2)	~48				
Defocus range (μm)	0.8-3				
Pixel size (Å)	1.062				
**Icosahedral reconstruction**					
Number of used micrographs	9,422			4,521	
Box size (pixels)	400 (bin2, pixel size 2.124Å)			(bin2, pixel size 2.124Å)	
Initial particle images (no.)	124,422			33,093	
Final particle images (no.)	76,360			27,353	
Symmetry imposed	I2			I2	
Map resolution (Å)	4.248			4.248	
FSC threshold	0.143			0.143	
**Sub-particle reconstruction**					
Box size (pixels)	300	300	300	300	300
Initial sub-particle images (no.)	915,547	915,547	310,662	43,720	1,583,481
Final sub-particle images (no.)	310,662	35,387	69,955	3,152	69,035
Symmetry imposed	C3	C3	C1	C5	C3
Map resolution (Å)	2.7	3.3	4.1	3.7	3.0
FSC threshold	0.143	0.143	0.143	0.143	0.143
**Refinement**					
Initial model used	None	7WHM		3JB3	
Model resolution (Å)	2.7	3.3		3.8	
FSC threshold	0.5	0.5		0.5	
CC (mask)	0.86	0.83		0.77	
Map sharpening *B* factor (Å^2)	-80	-120		-120	
Model composition					
Nonhydrogen atoms	9650	9284		17038	
Protein residues	1214	1170		2114	
Ligands	0	0		6	
B factors (Å^2)					
Protein	59.55	59.95		16.48	
Ligand				29.57	
R.m.s. deviations					
Bond lengths (Å)	0.006	0.009		0.003	
Bond angles (°)	0.626	0.796		0.546	
Validation					
MolProbity score	1.53	1.73		1.62	
Clashscore	6.25	7.53		8.37	
Poor rotamers (%)	0.00	0.10		0.00	
Ramachandran plot					
Favored (%)	96.86%	95.46%		97.01%	
Allowed (%)	3.14%	4.54%		2.94%	
Disallowed (%)	0.00%	0.00%		0.05%	
