# Supplementary material for: Multiple conformations of trimeric spikes visualized on a non-enveloped virus
Source: Nat Commun. 2022 Jan 27;13:550. doi: 10.1038/s41467-022-28114-0 (PMC8795420; doi:10.1038/s41467-022-28114-0)
Supplement: Supplementary file 3 — Description of Additional Supplementary Files [file 41467_2022_28114_MOESM3_ESM.pdf]

Description of Additional Supplementary Files

Legend for Supplementary Movies

Supplementary Movie 1. Animation showing a full CPV sporting both the closed and opened conformations of the trimeric spike. The video begins with a composite cryoEM density map consisting of the icosahedral capsid, in gray, and the two conformations of the trimeric spike from sub-particle reconstructions, in purple. This is followed by zoom-in views of the two conformations of the trimeric spike superposed with the atomic models, as ribbons, of the spike protein trimer.

Supplementary Movie 2. Morphing between the two conformations of the trimeric spike complex. Atomic models are first coloured by subunit, then by domain, to facilitate reader's familiarity of domain movement as described in the text.
