# Supplementary material for: Multiple conformations of trimeric spikes visualized on a non-enveloped virus
Source: Nat Commun. 2022 Jan 27;13:550. doi: 10.1038/s41467-022-28114-0 (PMC8795420; doi:10.1038/s41467-022-28114-0)
Supplement: Supplementary file 6 — Reporting Summary [file 41467_2022_28114_MOESM6_ESM.pdf]

nature research

Corresponding author(s): Z. Hong Zhou and Jingchen Sun

Last updated by author(s): Jan 4, 2022

Reporting Summary

Nature Research wishes to improve the reproducibility of the work that we publish. This form provides structure for consistency and transparency in reporting. For further information on Nature Research policies, see our Editorial Policies and the Editorial Policy Checklist.

Statistics

For all statistical analyses, confirm that the following items are present in the figure legend, table legend, main text, or Methods section.

n/a	Confirmed	
☐	☒	The exact sample size (*n*) for each experimental group/condition, given as a discrete number and unit of measurement
☐	☒	A statement on whether measurements were taken from distinct samples or whether the same sample was measured repeatedly
☒	☐	The statistical test(s) used AND whether they are one- or two-sided<br>*Only common tests should be described solely by name; describe more complex techniques in the Methods section.*
☒	☐	A description of all covariates tested
☒	☐	A description of any assumptions or corrections, such as tests of normality and adjustment for multiple comparisons
☒	☐	A full description of the statistical parameters including central tendency (e.g. means) or other basic estimates (e.g. regression coefficient) AND variation (e.g. standard deviation) or associated estimates of uncertainty (e.g. confidence intervals)
☒	☐	For null hypothesis testing, the test statistic (e.g. *F*, *t*, *r*) with confidence intervals, effect sizes, degrees of freedom and *P* value noted<br>*Give P values as exact values whenever suitable.*
☒	☐	For Bayesian analysis, information on the choice of priors and Markov chain Monte Carlo settings
☒	☐	For hierarchical and complex designs, identification of the appropriate level for tests and full reporting of outcomes
☒	☐	Estimates of effect sizes (e.g. Cohen's *d*, Pearson's *r*), indicating how they were calculated

Our web collection on statistics for biologists contains articles on many of the points above.

Software and code

Policy information about availability of computer code

Data collection	SerialEM 3.8
Data analysis	The EM softwares used: MotionCor 2, Ctffind 4.1.10, Ethan, Relion 3.0 and 3.1. Structure were built and refined using winCOOT 0.9.4 and Phenix 1.18.2. Structures were visualized using UCSF Chimera 1.14 and ChimeraX 1.1. Local resolution was estimated using Resmap 1.95. Potential sialic acid binding sites were predicted with Autodock Vina. Secondary structure predictions obtained from Phyre2.

For manuscripts utilizing custom algorithms or software that are central to the research but not yet described in published literature, software must be made available to editors and reviewers. We strongly encourage code deposition in a community repository (e.g. GitHub). See the Nature Research guidelines for submitting code & software for further information.

Data

Policy information about availability of data

All manuscripts must include a data availability statement. This statement should provide the following information, where applicable:

- Accession codes, unique identifiers, or web links for publicly available datasets
- A list of figures that have associated raw data
- A description of any restrictions on data availability

Cryo-EM density maps have been deposited in the Electron Microscopy Data Bank (EMDB) under accession numbers EMD-32504 (in situ structure of the closed spike), EMD-32505 (in situ structure of the opened spike), EMD-32506 (in situ structure of the trimeric spike interact with pentameric turret), EMD-32507 (in situ structure of the pentameric turret after the trimeric spike detaches), and EMD- 32508 (detached spike). Accession codes for atomic models deposited in the Protein Data Bank are 7WHM (closed spike), 7WHN (opened spike), and 7WHP (turret protein after the trimeric spike detaches).

Field-specific reporting

Please select the one below that is the best fit for your research. If you are not sure, read the appropriate sections before making your selection.

☒ Life sciences ☐ Behavioural & social sciences ☐ Ecological, evolutionary & environmental sciences

For a reference copy of the document with all sections, see nature.com/documents/nr-reporting-summary-flat.pdf

Life sciences study design

All studies must disclose on these points even when the disclosure is negative.

Sample size	No statistical methods were used to predetermine sample size. 1mL CPV polyhedra suspension was used each time for CPV purification, which can provide enough sample for cryo-EM analysis. For q-CPV sample, icosahedral reconstructions were calculated from 9,422 images (76,360 particles) for structure of q-CPV capsid, sub-particle reconstruction were calculated from 310,662 sub-particles for structure of closed spike, and 35,387 sub-particles for structure of opened spike, and 69,955 sub-particles for structure of closed spike interacts with pentameric turret. For SA-CPV sample, 4,521 images (27,353 particles) for structure of SA-CPV capsid, sub-particle reconstruction were calculated from 3,152 sub-particles for structure of pentameric turret after spike detaches, single particle reconstruction were calculated from 69,035 particles for structure of detached closed spike. The data size is comparable to other studies in this field. The number of particles used for each final map is sufficient to obtain reliable classification and reconstruction results by cryo-EM.
Data exclusions	For cryo-EM analysis, particles that do not belong to the class of interest or have poor qualities based on well established cryoEM principle were excluded after rounds of 2D and 3D classification. This is standard practice required to obtain high resolution cryo EM structure of the class of interest.
Replication	Individual cryo-EM dataset has been processed separately and give rise to similar reconstruction. Virion-liposome binding assay was repeated two times independently with similar results.
Randomization	For cryo-EM, datasets were randomly split into two half for refinements. For the biochemical experiments, randomization is not applicable to our study, because no grouping was needed.
Blinding	For cryo-EM, datasets were automatically split into two half in Relion, blinding is not feasible. For the biochemical experiments, blinding is not applicable as no grouping was needed for this study.

Reporting for specific materials, systems and methods

We require information from authors about some types of materials, experimental systems and methods used in many studies. Here, indicate whether each material, system or method listed is relevant to your study. If you are not sure if a list item applies to your research, read the appropriate section before selecting a response.

Materials & experimental systems

n/a	Involved in the study
☒	☐ Antibodies
☒	☐ Eukaryotic cell lines
☒	☐ Palaeontology and archaeology
☒	☐ Animals and other organisms
☒	☐ Human research participants
☒	☐ Clinical data
☒	☐ Dual use research of concern

Methods

n/a	Involved in the study
☒	☐ ChIP-seq
☒	☐ Flow cytometry
☒	☐ MRI-based neuroimaging
